# The Week in the Courts

**Published:** 1911-04-29

**Authors:** 


### THE WEEK IN THE COURTS.

### MEDICAL PRACTITIONER'S ACTION FOR SLANDER.

At Reading, before Mr. Justice Eldon Bankes, Dr. H. S. Challenor, of Abingdon, brought an action against Miss Annie Anson for slander. The ground of the action was a conversation which Dr. Challenor's sister had overheard in a railway carriage; the defendant and others were travelling in the same compartment and the defendant was said to have made some disparaging remarks about the plaintiff, who is one of the medical staff at the Abingdon Cottage Hospital, to the effect that she could not find his name on any Medical List, and that at the hospital they could not do the smallest operation. Dr. Challenor said he brought the action "in order to stop a woman's tongue." The defendant denied that she made any imputations. The jury found for the plaintiff with a farthing damages, and costs to follow the action. Similar actions against Miss Anson were entered by Dr. Woodward and Dr. Martin, both of Abingdon, and counsel agreed to judgment for similar damages, but in these cases the costs were disallowed.

### AN UNREGISTERED DENTIST.

At Bolton Police Court, J. H. Mawson was summoned for using the title of surgeon-dentist and thereby infringing the Dentists Act. Defendant, it was alleged, had used bill-heads on which his name appeared, together with the title. For the defence it was stated that defendant had previously practised in the Isle of Man where, previous to the Act coming into force last year, he was entitled to use the description. The use of the title on the bills after coming to Bolton was an oversight. The Bench decided to impose a fine of four guineas and costs, but on defendant's solicitor submitting that the Medical Register had not been produced to show that defendant was not a legally qualified medical practitioner, the magistrates dismissed the case on that ground.

